# Novel Application of NIR Spectroscopy for Non-Destructive Determination of ‘Maraština’ Wine Parameters

**DOI:** 10.3390/foods11081172

**Published:** 2022-04-18

**Authors:** Jasenka Gajdoš Kljusurić, Ana Boban, Ana Mucalo, Irena Budić-Leto

**Affiliations:** 1 Faculty of Food Technology and Biotechnology, University of Zagreb, Pierottijeva 6, 10 000 Zagreb, Croatia; jgajdos@pbf.hr; 2 Institute for Adriatic Crops and Karst Reclamation, Put Duilova 11, 21 000 Split, Croatia; ana.mucalo@krs.hr (A.M.); irena.budic-leto@krs.hr (I.B.-L.)

**Keywords:** wine, ‘Maraština’, L*, a*, b*, chroma, NIR spectroscopy, chemometrics

## Abstract

This study investigates the colour and standard chemical composition of must and wines produced from the grapes from *Vitis vinifera* L., ‘Maraština’, harvested from 10 vineyards located in two different viticultural subregions of the Adriatic region of Croatia: Northern Dalmatia and Central and Southern Dalmatia. The aim was to explore the use of NIR spectroscopy combined with chemometrics to determine the characteristics of Maraština wines and to develop calibration models relating NIR spectra and physicochemical/colour data. Differences in the colour parameters (L*, a*, hue) of wines related to the subregions were confirmed. Colour difference (ΔE) of must vs. wine significantly differed for the samples from the Maraština grapes grown in both subregions. Principal component regression was used to construct the calibration models based on NIR spectra and standard physicochemical and colour data showing high prediction ability of the 13 studied parameters of must and/or wine (average R^2^ of 0.98 and RPD value of 6.8). Principal component analysis revealed qualitative differences of must and wines produced from the same grape variety but grown in different subregions.

## 1. Introduction

The chemical composition of wine is highly influenced by the grape variety, the specific conditions of the vineyard location associated with both climate and soil characteristics, as well as the vinification technology used to produce the wine [1]. These factors give each wine its special characteristics, which for most wine-producing countries in Europe are defined by the geographical indication of origin [2]. In accordance with Regulation (EU) No 1308/2013, Croatian wines made in two different viticultural areas of Dalmatia, Northern Dalmatia and Central and Southern Dalmatia, can have protected designation of origin (PDO). The wines produced in coastal viticultural subregions are influenced by different production methods, where terroir has an important impact. Terroir is a viticultural area where vine interacts with the environment and agronomic practices in vineyards. Many factors are included in terroir expression, such as cultivar; the geographic position, soil, and climate conditions of a vineyard; and vineyard management. On this basis, it could be proposed that if the grape composition is marked by chemical fingerprints from a given terroir, wines made from these grapes should also reflect the relevant fingerprints [3]. The impacts of climate on vine development and grape ripening are exerted primarily through rainfall, irradiation and air temperature [4]. Solar radiation and temperature regime strongly affect phenology and grape maturity [5]. The soil influences vine and grape primarily through soil temperature, water supply and mineral supply.

Analytical methods used for the characterization of wines according to geographic origin, such as high-performance liquid chromatography, gas chromatography, inductively coupled plasma spectrometry and atomic absorption spectroscopy, demand expensive instruments as well as different preparation and extraction procedures, and the detection techniques are time-consuming [6,7]. On the other hand, near-infrared (NIR) spectroscopy is a simple, rapid and non-destructive method for the detection of chemical composition concerning the relative proportions of C-H, N-H and O-H molecular bonds in the NIR spectral region (750–2500 nm) that behaves as a fingerprint of the sample [8]. Several articles have reported the potential of NIR spectroscopy to measure a number of oenological parameters, such as alcohol content, pH, volatile acids, organic acids and reducing sugars in grapes and wine [9,10]. The pH and organic acids can be predicted using NIR transmission spectra in the wavelength range 700–1060 nm [11], and total soluble solids [12] at wavelengths from 900 to 2200 nm. Furthermore, NIR spectroscopy can be a powerful tool for the traceability of geographic origin and variety. NIR and chemometric methods were used as rapid techniques to classify subzones in a designation of origin Rías Baixas [13], and in Tempranillo wines from Spain and Australia [14].

As Wildenradt and Stafford concluded in 1977 when investigating the colour of white wine and white grape juice using a colorimeter, the acceptance of white wines is highly influenced by the colour of the wine (the same is true for foods as well) [15,16]. Therefore, for wines produced from grapes of the Maraština variety, we monitored the colour and its similarities and differences, depending on the place of harvest. White wine colour intensity is related to the maceration treatment [17], whereby the fermentative addition of overripe seeds leads to a significantly higher content of procyanidins than traditionally macerated wines, also resulting in an effect on colour quality and stability [18]. A number of different available data sets provide excellent inputs for chemometric analysis, that is, the use of mathematical, statistical and other methods to provide maximal information from relevant chemical data [19].

‘Maraština’, a variety of grape from *Vitis vinifera* L., is an autochthonous variety mostly cultivated in the Adriatic coastal region of Croatia (Dalmatia) [20]. In the past, this variety was famous for Prošek dessert wine production. The wines of this cultivar are very diverse regarding colour and quality. Although there are several Maraština producers on the Croatian market, there is a lack of research on this wine’s chemical composition. Determination of geographic origin is a good indicator of the authenticity of wine, and NIR spectroscopy has previously been used to test wine authenticity.

The aim of this study was to explore the use of NIR spectroscopy, combined with chemometrics, to (i) characterize Maraština grapes and wines produced from grapes derived from two subregions of Dalmatia (Northern Dalmatia and Southern and Central Dalmatia) regarding the standard physicochemical parameters and colour data; and (ii) use the chemometric approach to develop calibration models for correlating the NIR spectra and physicochemical/colour data of must with the physicochemical/colour data of wine.

## 2. Materials and Methods

### 2.1. Grape Samples

**Figure 1:**
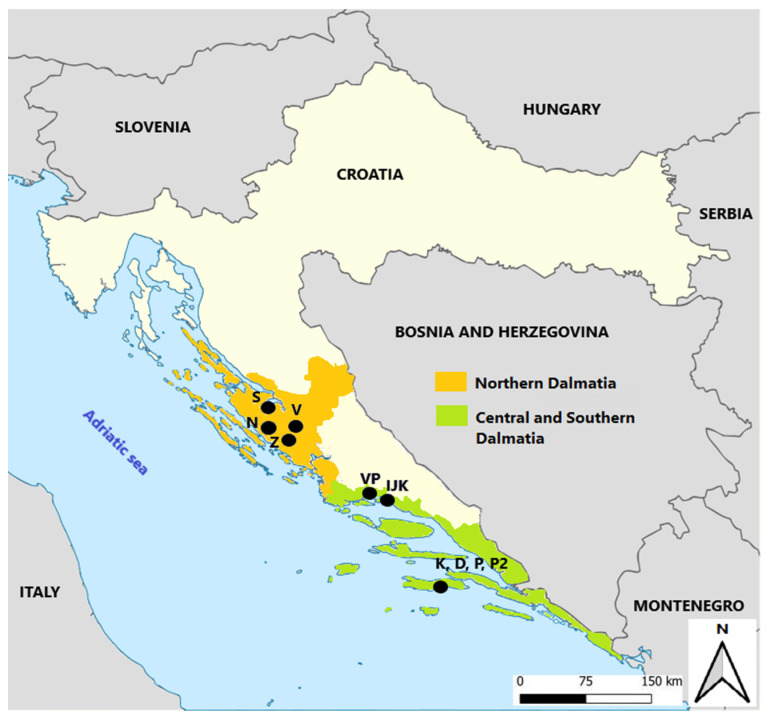
Geographic locations of 10 vineyards in the vine growing subregions: Northern Dalmatia (N—Polača, S—Smilčić, Z—Stankovci, V—Vukšić), Central and Southern Dalmatia (IJK—Institute for Adriatic Crops and Karst Reclamation, VP—Kaštela; on the island of Korčula: K—Kruševo, D—Dračevica, P—Prapatna 1, P2—Prapatna 2).

Healthy grape samples from undamaged vines were collected from the germplasm repository of the Institute for Adriatic Crops and Karst Reclamation at Split and nine commercial vineyards during the 2021 vintage (Figure 1).

The vineyards are located along the Croatian coastal area in the two different viticultural subregions (Table S1). During September 2021, a total of 10 samples of Maraština grapes were collected in triplicate at technological maturity. In detail, the sampling plan consisted of three randomized blocks in the middle of each vineyard. A block was formed by one row of vines. One sample for each block was composed of nine well-exposed bunches collected from three different vines from the top, middle and end of the row. The samples (around 3 kg each) were collected and transported to the laboratory in a cool bag.

### 2.2. Experimental Wine Production

Grapes were crushed manually and homogenized to obtain fresh must for chemical analysis. The musts were sulphited with potassium metabisulphite to give a concentration of total SO_2_ in wine of approximately 50 mg/L. Wines were produced by spontaneous alcoholic fermentation, without added inoculated yeast. All fermentations were performed in 500 mL Erlenmeyer flasks protected from light with aluminium foil. The fermentations were carried out at 20 °C. The fermentation progress was monitored daily by measuring the sugar content and fermentation temperature. Samples from the end of the fermentation were taken for analysis of standard wine physico-chemical parameters.

### 2.3. Chemical Analysis of Fresh Must and Wines

The content of total soluble solids (TSS) in must, °Brix, was measured using a refractometer Hi 96814 (Hanna Instruments, Woonsocket, RI, USA). The pH was measured using a Titrino 718 pH meter (Metrohm, Herisau, Switzerland), and total acidity (TA) was determined by titrating with 0.1 M sodium hydroxide solution to a pH end-point of 7. Basic chemical parameters of fresh must were determined according to the reference OIV methods for wine analysis (OIV, 2021) in a laboratory accredited according to HRN EN ISO/IEC 17025. Wines were analysed by an FTIR Lyza 5000 Wine (Anton Paar GmbH, Graz, Austria) to determine the oenological parameters: pH, relative density (20/20 °C), alcohol (vol %), total dry extract (g L^−^^1^), reducing sugars (g L^−^^1^), total acidity expressed in (g L^−^^1^) of tartaric acids and volatile acidity expressed in (g L^−^^1^) of acetic acids. 

### 2.4. Colour Measurement

For colour measurement we used a PCE-CSM3 colorimeter (PCE Instruments, Meschede, Germany) with prior white plate calibration. The instrument allows the measurement of the following parameters: (i) Hunter’s colour coordinates (lightness, L*; the range from green to red, a*; and the range from blue to yellow, b*); (ii) relative saturation, chroma and (iii) the angle of the hue to describe the colour changes from must to wine. To compare the values depending on the original vineyard, the total colour change, ΔE, was determined using the following equation:

$$\Delta E\;=\sqrt{{\left(L^{*}-{\;L}_{0}\right)}^{2}+{\left(a^{*}-{\;a}_{0}\right)}^{2}+{\left(b^{*}-{\;b}_{0}\right)}^{2}}$$

where L*, a* and b* were determined for wines, whereas L_0_, a_0_ and b_0_ were determined for musts. Three parallel measurements (technical replicates) were performed for each sample, and the results are presented as the mean value ± standard error.

### 2.5. Near-Infrared Spectroscopy

A Control Development Inc. NIR spectrometer, NIR-128-1.7-USB/6.25/50 μm, was used for the NIR spectroscopy of must and wine samples. All samples were scanned in a cuvette placed in a light-insulated holder. Measurements were performed in the near-infrared wavelength range from 904 to 1699 nm at ambient temperature (path length 1 nm) representing the range of the instrument used. The device was controlled, and the data were processed, by the Control Development software Spec32. For each wine and must sample (10 vineyards in triplicate) the average NIR scan was calculated.

### 2.6. Data Analysis

All physicochemical and colour data of musts and wines were analysed by ANOVA. We investigated potential outliers in all the observed parameters using boxplots (Figure S1). No outliers were identified in the must data.

The chemometric approach started with the application of principal component analysis (PCA) in order to determine the qualitative similarities or differences of the samples from different regions (Northern Dalmatia and Central and Southern Dalmatia). The sets of spectral data (a single ‘data’ file for each sample scan) were exported from Spec32 software into Excel and then into Unscrambler software (version X 10.2; CAMO ASA, Oslo, Norway) for pre-treatment of NIR scans for the calibration models, which were obtained. Calibration models based on the physicochemical properties, colour data and NIR spectra were developed using partial least squares regression (PLS) for all the studied parameters (Table 1). Principal components regression (PCR) was applied to calculate the principal components and then use some of these components as predictors in a linear regression model fitted using the typical least squares procedure Gen. In order to determine the best model results for each compound, the coefficient of determination (R^2^), root mean square error (RMSE) values and residual predictive deviation (RPD) were used.

## 3. Results and Discussion

All wines from the 10 locations tested to the physicochemical parameters and they had low reducing sugars (below 1.0 g L^−^^1^) and volatile acidity (0.45 and 0.46 g L^−^^1^), indicating good spontaneous fermentation activity by natural yeasts in all samples (Table 1). The alcohol content in Maraština wines was between 12.65 and 13.29 vol %, without statistically significant differences among the subregions. This could be due to large variation in the content of total soluble solids (TSS) derived from diverse grape sample composition and vineyard influences, as can be seen from Table 1. Total acidity content was significantly higher in Maraština wine produced from grapes derived from the Northern Dalmatia subregion, indicating the importance of geographic position and climate [21]. Additionally, Maraština wine from Northern Dalmatia was characterized by significantly higher total dry extract (21.4 g L^−^^1^) than wine from Central and Southern Dalmatia (20.1 g L^−^^1^). 

**Figure 2:**
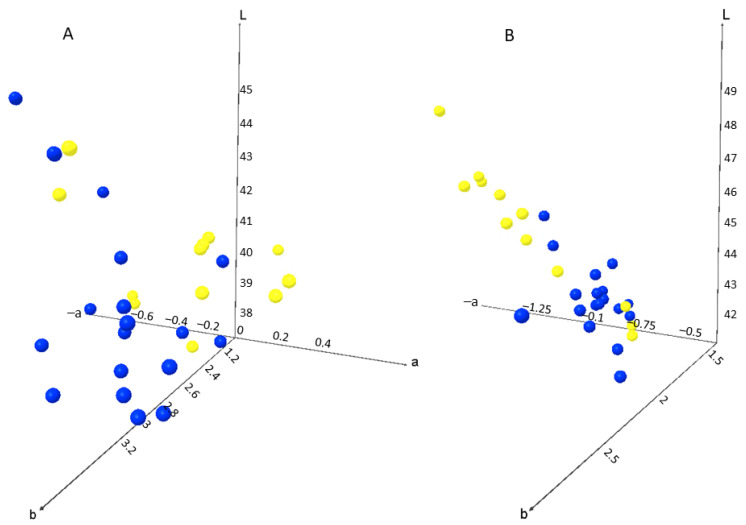
L*a*b* colorimetry for musts (**A**) and wines (**B**) from two Dalmatian subregions (blue dots represent data from Central and Southern Dalmatia and yellow dots represent data from Northern Dalmatia).

All parameters related to the colour (L*, a*, b*, chroma and hue) are direct outputs of the used instrument. Comparing the colour parameters, the lowest L* value was measured for must produced from the grapes harvested in vineyard K (Table S3) (L* = 38.8 ± 0.6), whereas the highest L* value was found in grapes from vineyard VP (L* = 43.6 ± 1.2); both vineyards are in Central and Southern Dalmatia. The darkest wines were from vineyards D and N, whereas the lightest wines were produced from the grapes harvested in the Northern Dalmatian vineyards V (L* = 46.9 ± 1.5) and Z (L* = 46.7 ± 0.1). It is important to point out that for both must and wine, the parameter b* (−b: blue to b+: yellow) did not show a significant deviation among the vineyards (Supplementary Tables S2 and S3). In the case of must, the values for the parameter a* indicated the presence of colours from green to red (−a to +a), but in wine this effect was lost, and the colours were only in the green spectrum (−a values) (Figure 2). As outliers were identified the total acidity (>7 g/L), relative density (>0.9950 20/20 °C), alcohol (<10 vol%) and all reducing sugar (RS) values higher than 2. Outliers for the colour parameters were a* < −1 and b* and chroma > 3. The parameter b* remained in the yellow spectrum (+b values) for must and wine, with the values of 2.6–3.2 for must and 1.8–2.2 for wine. Relative saturation was higher in the musts obtained from the grapes harvested in Central and Southern Dalmatia compared with those from Northern Dalmatia, whereas all wines had lower relative saturation values than musts and the highest values were measured for wines from Northern Dalmatia subregion (Table S3). Rivero [18] reported that the colour parameters of white wine during the vinification stage showed the same trend in L* (slightly raising in the stabilization stage and bottling), but their relative saturation showed an inverse relationship with L* and hue. The output values for the tone (hue, its unit is the sexagesimal degree (°)) are less than 90° when parameters a* and b* are positive, and between 90° and 180° when the parameter a* is negative (the case in our study for all wines and only for a few musts). If both parameters are negative, the tone will be in the range of 270°and 360° and between 180° and 270° if parameter b* is negative [22]. 

In our study, the angle of the hue was lower in the must compared to wine, and was positively correlated (r = 0.316) only with the lightness (L*); it was inversely proportional to the other observed colour parameters (r < 0). Castellanos and co-workers also confirmed significant changes in the parameters of wine colour, depending on the temperature at which the wine was stored [23].

**Figure 3:**
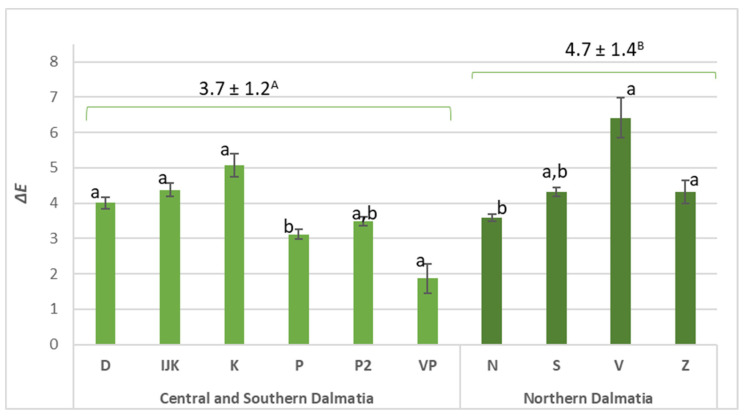
Colour changes (ΔE) between musts and their wines. Different letters denote significant differences among the vineyards (means ± SEs), and capital letters indicate regional differences (both at *p* < 0.05).

**Figure 4:**
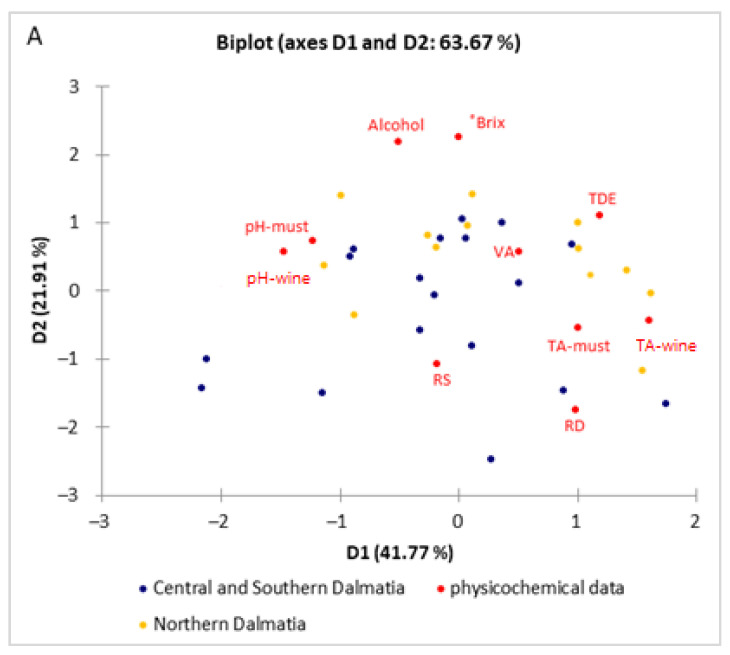

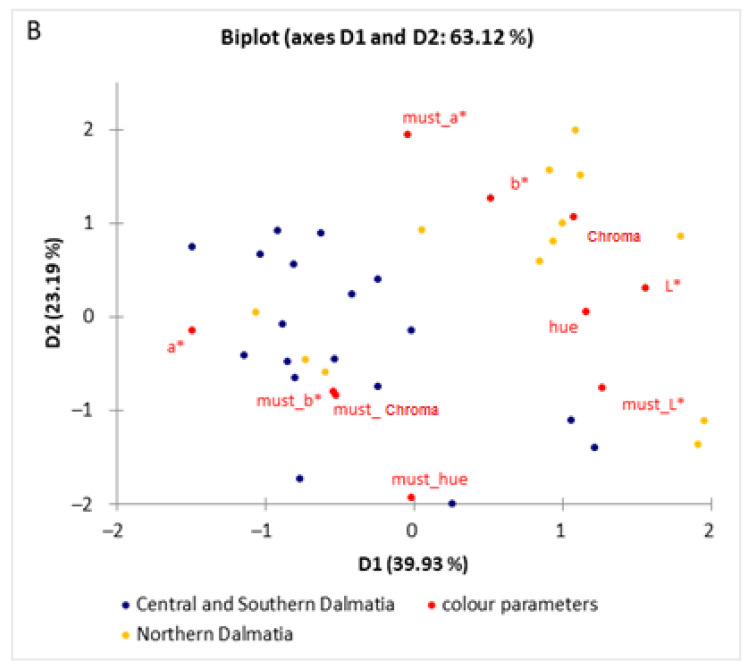
Qualitative observation by use of principal component analysis of must and wine physicochemical data (**A**) and colour parameters (**B**) produced from grapes of the Maraština variety grown in 10 different vineyards in two Dalmatian subregions.

Observing ΔE values for all 10 vineyards (Figure 3) in Dalmatian subregions, the colour change between must and wine was above the threshold (ΔE 2–10: perceptible at a glance), with one exception (VP) just below the ΔE value of 2 (ΔE_VP_ = 1.9 ± 1.3). Dominant colour change was detected for must and wine produced from grapes harvested in vineyard V (ΔE_V_ = 6.4 ± 1.7). The colour in the Northern Dalmatian subregion was significantly different (ΔE_ND_ = 4.7 ± 1.4; *p* = 0.0169). The addition of overripe seeds from white grape by-products during wine fermentation also affects the colour, as presented by Rivero [18]. To observe these qualitative differences for physicochemical and colour parameters simultaneously in two defined regions, a principal component analysis was performed (Figure 4).

**Figure 5:**
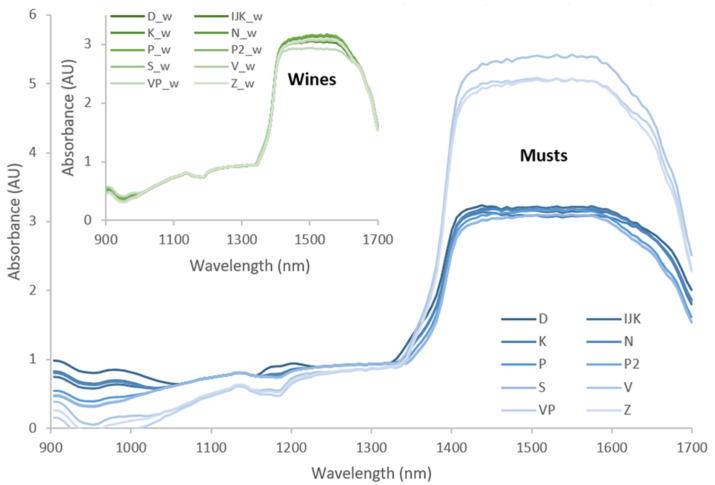
NIR spectra of musts and wines produced from Maraština grapes harvested in 10 different vineyards.

It is evident from the PCA analysis that the colour parameters, depending on the grape growing region, differed to a much lesser extent in must (significant difference for chroma and hue parameters), while wines differed in brightness (L*), green colour (−a*) and tone (hue parameter). Considering the measured physicochemical parameters (Figure 4A), there was an unclear separation of samples from the two observed regions. With respect to the colour parameters (Figure 4B), the samples from Central and Southern Dalmatia (left side) were more separated when they were compared with the samples from Northern Dalmatia. These findings were certainly an incentive to continue looking for potential links in the chemical composition of must and wine, and the next step was to record the near-infrared spectra (Figure 5).

NIR spectroscopy is known as a non-invasive and rapid method. Chemometric tools are numerous, and the skill of the person processing and combining the chemical parameters is crucial. The scanned near-infrared spectra included the region of electromagnetic spectrum between 904 nm and 1699 nm, which is related to vibration and combination of the fundamental C-H, O-H and N-H bonds in organic molecules [13]. The NIR spectra of wine are essentially composed of large sets of overtones and combination bands, but many of the absorption peaks of water overlap with the absorption peaks of the other molecular species present in the grape [24], must and wine, making the distinguishing task difficult. Therefore, in order to assign specific absorption bands to specific functional groups, multivariate statistical techniques (i.e., chemometrics) are applied to extracting information from the spectral data [25]. This involves regression techniques coupled with spectral pre-processing [26] and the baseline evaluation method, and the second derivative spectrum is often used to assist in the identification of further absorption bands, particularly small and/or overlapping absorption peaks not resolvable in original raw spectra [27,28,29]. The most informative parts of spectra can be discerned by application of chemometric techniques [30]. In order to move from qualitative to quantitative observation of data, and to predict the expected parameters of wine based on the input parameters of must and/or wine, an additional chemometric technique (principal component regression, PCR) was applied to obtain calibration models of the NIR spectra related to all physicochemical data and colour parameters. The calibration models were developed using full cross-validations. Spectral data were pre-treated before modelling by using (i) smoothing: moving average, Gaussian filter, median filter and Savitzky–Golay; (ii) normalization; (iii) derivative: gap, gap-segment, and Savitzky–Golay (1st derived or 2nd derived); (iv) baseline; (v) standard normal variate (SNV); and (vi) de-trending. The input matrix of modelling data consisted of 66 rows and 813 columns, of which 796 columns represented the NIR spectral data, with as many models as there were data on the chemical composition and colour of must and/or wine. The combination of SNV and SG second derivation proved to be the most efficient spectral pre-processing procedure, followed by further modelling. Applying the coefficients of determination and their qualitative characteristics known as the Chaddock scale [31], the majority of R^2^ values indicated strong relationships (R^2^ of 0.7–0.9) between the observed input vs. predicted output wine parameters. Outside this range were the R^2^ values for the prediction of wine volatile acidity (VA) and the colour parameter b*, for which the relationship between the observed input and predicted output parameters was not strong, whereas reduced sugars (RS) and colour parameter hue showed a significant but moderate relationship (R^2^ ≥ 0.3–0.5). Lesser relationships were expected for the parameters that depended on other variables (VA on fermentation and RS on yeast fermentation activity) [30].

The statistical parameters used to assess the success of the calibration are R^2^, RMSE and residual predictive deviation (RPD) [16]. The modelling started with determination of the success of the calibration of measured data (colour and physicochemical parameters) based solely on the NIR spectra of must and wine (must data related with must spectra and wine data related with wine spectra). Finally, we examined the relationship of the must inputs (NIR spectra, basic physicochemical data and colour data) to predict the expected physicochemical and colour parameters for wine, regardless of the region.

The RPD value (residual predictive deviation) is defined as the ratio between the standard deviation of the population (SD) and the standard error of cross-validation (SECV); values of RPD greater than three are considered fair and are recommended for screening purposes, whereas an RPD value greater than 5 is good, making the model suitable for quality control [32].

The vast majority of the RPD values calculated in the present report (Table 2) were higher than three, confirming the good performance of the developed models. The high RPD values were positively correlated with the higher values of the coefficient of determination, and with lower RMSE values. In most cases (i.e., 7 out of the 12 studied wine parameters), advantage was found in the models developed using must data M (must–wine). Lower RPD values (<3) were detected for total acidity, total dry extract, reducing sugars and the two parameters of colour (lightness and hue angle). Charts showing the agreement between the measured parameters for wine and the values obtained by PLS models are given in Figure S2. The good agreement and acceptability of the model for qualitative prediction is evident. Similarly, in the study of De Bei and co-workers [33], the prediction of total non-structural carbohydrate concentration in grapevine trunk and leaf tissues was successful using near-infrared spectroscopy. It needs to be emphasized that the chemometric approach used in the present study to correlate the NIR spectra and physicochemical/colour data of must and relate them with the wine physicochemical/colour data can be considered novel; it allowed good predictions to be obtained for the parameters tested. With more input data, the final expectations could be influenced and possibly corrected. Such models give insight into what to expect in wine samples, as the final product.

## 4. Conclusions

Based on the physicochemical parameters, there was an unclear separation of samples from the two observed regions. Furthermore, colour parameters of the must and wine samples from Northern Dalmatia differed from those from Central and Southern Dalmatia for the majority of the observed parameters, whereas the colour difference (ΔE) of must vs. wine differed significantly for Maraština grapes grown in Northern Dalmatia. This study showed that NIR spectroscopy can be used to determine expected Maraština wine parameters only by knowing the must characteristics and NIR spectra. This approach is non-destructive and does not require prior sample treatment, and thus represents a simple and rapid way to gain important information about samples under investigation, allowing qualitative and quantitative differentiation based on chemometric tools.

## Figures and Tables

**Table 1:** Standard physicochemical and colour data (mean ± standard deviation) of must and wines produced from grapes of the Maraština variety grown in 10 different vineyards. Different letters in the same row denote significant differences at *p* < 0.05.

Parameters		Central and Southern Dalmatia	Northern Dalmatia
**Standard chemical composition**	***Must***		
	°Brix	20.9 ± 2.0 ^a^	22.2 ± 1.5 ^b^
	pH	3.55 ± 0.08 ^a^	3.51 ± 0.12 ^a^
	TA (g L^−1^)	4.79 ± 1.07 ^a^	4.46 ± 0.62 ^b^
	***Wine***		
	pH	3.45 ± 0.09 ^a^	3.43 ± 0.11 ^a^
	TA (g L^−1^)	5.58 ± 0.50 ^a^	5.72 ± 0.51 ^a^
	VA (g L^−1^)	0.46 ± 0.06 ^a^	0.45 ± 0.05 ^a^
	RD (20/20 °C)	0.9912 ± 0.0019 ^a^	0.9910 ± 0.0015 ^a^
	Alcohol (vol %)	12.65 ± 1.69 ^a^	13.29 ± 1.11 ^a^
	TDE (g L^−1^)	20.1 ± 1.7 ^a^	21.4 ± 1.7 ^b^
	RS (g L^−1^)	0.8 ± 0.8 ^a^	0.1 ± 0.1 ^b^
**Colour parameters**	***Must***		
	L*	40.19 ± 1.81 ^a^	40.89 ± 1.38 ^a^
	a*	−0.07 ± 0.23 ^a^	0.04 ± 0.27 ^a^
	b*	3.05 ± 0.21 ^a^	2.76 ± 0.23 ^b^
	Chroma	3.06 ± 0.21 ^a^	2.77 ± 0.23 ^b^
	Hue	91.58 ± 4.51 ^a^	89.11 ± 5.56 ^a^
	***Wine***		
	L*	43.56 ± 0.91 ^a^	45.36 ± 1.92 ^b^
	a*	−0.67 ± 0.10 ^a^	−0.91 ± 0.25 ^b^
	b*	1.96 ± 0.24 ^a^	1.99 ± 0.16 ^a^
	Chroma	2.07 ± 0.22 ^a^	2.20 ± 0.20 ^a^
	Hue	109.02 ± 3.77 ^a^	114.34 ± 5.65 ^b^

TA—total acidity expressed as tartaric acid; VA—volatile acidity expressed as acetic acid; RD—relative density; TDE—total dry extract; RS—reducing sugars; L*—lightness; a*—the range from green to red; b*—the range from blue to yellow; chroma—colour intensity; hue—colour changes from must to wine.

**Table 2:** Calibration of wine parameters based on the physicochemical data and colour of must.

Calibrated/Predicted Parameters	M (Must–Must)			M (Wine–Wine)			M (Must–Wine)		
	R^2^	RPD	RMSE	R^2^	RPD	RMSE	R^2^	RPD	RMSE
°Brix	0.993	2.403	0.876	-	-	-	-	-	-
pH	0.998	5.154	0.022	0.980	12.033	0.104	0.990	4.773	0.073
TA (g L^−1^)	0.984	11.414	0.654	0.963	1.144	0.806	0.968	1.323	0.750
VA (g L^−1^)	-	-	-	0.920	6.064	0.103	0.961	12.276	0.073
RD (20/20 °C)	-	-	-	0.966	9.603	0.002	0.994	19.090	0.001
Alcohol (vol%)	-	-	-	0.996	4.820	0.548	0.999	22.862	0.184
TDE (g L^−1^)	-	-	-	0.937	2.297	2.736	0.952	2.091	2.400
RS (g L^−1^)	-	-	-	0.989	4.489	0.393	0.963	1.234	0.749
L*	0.990	2.030	0.905	0.999	12.621	0.127	0.952	0.807	1.951
a*	0.988	10.566	0.155	0.998	4.114	0.498	0.968	4.742	0.208
b*	0.999	15.952	0.043	0.998	5.331	0.038	0.992	19.003	0.102
Chroma	0.999	5.737	0.045	0.997	3.450	0.061	0.999	10.475	0.020
Hue	0.991	1.886	2.689	0.999	10.058	0.506	0.956	2.532	6.033

TA—total acidity expressed as tartaric acid; VA—volatile acidity expressed as acetic acid; RD—relative density; TDE—total dry extract; RS—reducing sugars; “-”—not determined in the must; L*—lightness; a*—the range from green to red; b*—the range from blue to yellow; chroma—colour intensity; hue—colour changes from must to wine.

## Data Availability

Data is contained within the article and also available on request.

## Supplementary Materials

The following supporting information can be downloaded at: https://www.mdpi.com/article/10.3390/foods11081172/s1, Figure S1: Use of boxplot to identify outliers in the observed data set; Figure S2: Predicted values for the studied compounds (calculated from the developed models) vs. experimental data; Table S1: Global position of vineyards and wine subregions; Table S2: The physicochemical data of must and wines produced from grapes of the ‘Maraština’ variety grown in 10 different vineyards in two Dalmatian subregions; Table S3: Colour parameters of must and wine.
