# Supplementary material for: Novel Application of NIR Spectroscopy for Non-Destructive Determination of ‘Maraština’ Wine Parameters
Source: Foods. 2022 Apr 18;11(8):1172. doi: 10.3390/foods11081172 (PMC9025932; doi:10.3390/foods11081172)
Supplement: Supplementary file 1 [file foods-11-01172-s001.zip › foods-1671421-supplementary.pdf]

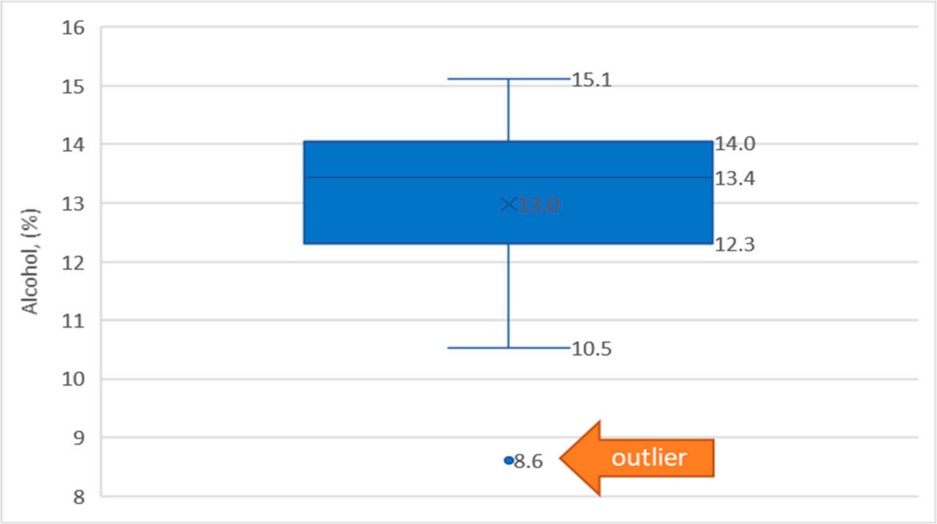

Figure S1. Use of boxplot to identify outliers in the observed data set (e.g., ethanol (vol %) in wine), where the value of 10.5 vol% alcohol was the minimum (lowest) value, excluding outliers and 15.1 vol% alcohol was the maximum (greatest) value, excluding outliers, which are located within Q1±1.5 x interquartile range.

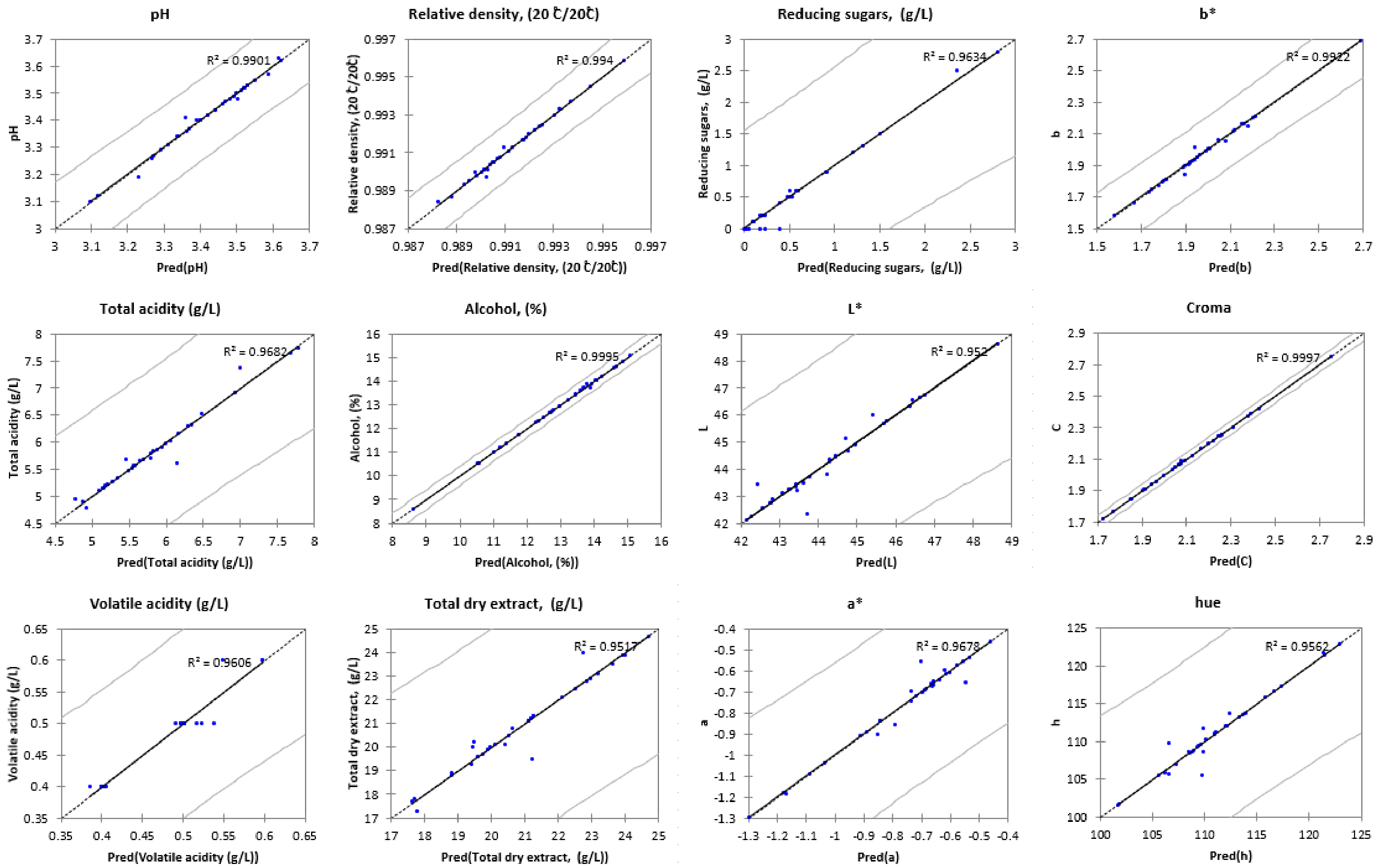

Figure S2. Predicted values for the studied compounds (calculated from the developed models) vs. experimental data. Blue circles represent the calibration data for wines calculated from PCR models based on must data in the range of confidence interval (grey lines).

Table S1. Geographical position of vineyards and wine subregions.

Name of Vineyard	Sample Abbreviation	Trellis System	Wine Subregion	Vineyard Geographical Position
Dračevica	D	bilateral cordon	Central and Southern Dalmatia	42°55′36″ N, 16° 54′0″ E
Institute for Adriatic Crops and Karst Reclamation	IJK	bilateral cordon	Central and Southern Dalmatia	43°30′35″ N, 16°29′85″ E
Kruševo	K	bilateral cordon	Central and Southern Dalmatia	42° 55′20″ N, 16° 53′54″ E
Prapatna 1	P	bilateral cordon	Central and Southern Dalmatia	42°54′51″ N, 16°54′58″ E
Prapatna 2	P2	bilateral cordon	Central and Southern Dalmatia	42° 54′50″ N, 16° 54′52″ E
Kaštel Kambelovac	VP	bilateral cordon	Central and Southern Dalmatia	43° 33′34″ N, 16° 22′32″ E
Polača	N	bilateral cordon	Northern Dalmatia	44° 0′28″ N, 15° 29′41″ E
Smilčić	S	bilateral cordon	Northern Dalmatia	44° 7′23″ N, 15° 28′52″ E
Vukšić	V	bilateral cordon	Northern Dalmatia	43° 56′37″ N, 15° 43′46″ E
Stankovci	Z	bilateral cordon	Northern Dalmatia	43° 57′20″ N, 15° 43′6″ E

Table S2. The physicochemical data of must and wines produced from grapes of the ‘Maraština’ variety grown in 10 different vineyards in two Dalmatian subregions. Different letters in the same column represent significant differences at $p < 0.05$. Means ± SD (n = 3).

Vineyard	Must			Wine						
	°Brix	pH	TA (g L^{-1})	pH	TA (g L^{-1})	VA (g L^{-1})	RD (20/20˚C)	Alcohol (vol %)	TDE (g L^{-1})	RS (g L^{-1})
**D**	18.1 ± 0.6^{a}	3.48 ± 0.07^{a}	5.72 ± 0.67^{a}	3.35 ± 0.03^{b}	6.31 ± 0.55^{a}	0.5 ± 0.00^{a}	0.9947 ± 0.0011^{a}	9.9 ± 1.12^{a}	20.83 ± 1.45^{a}	1.63 ± 1.11^{a}
**IJK**	22.4 ± 0.5^{b}	3.61 ± 0.04^{b}	6.41 ± 0.18^{a}	3.50 ± 0.03^{c}	5.67 ± 0.22^{a}	0.47 ± 0.06^{a}	0.9912 ± 0.0006^{b}	13.37 ± 0.78^{c}	22.13 ± 1.42^{a}	0.27 ± 0.12^{a}
**K**	21.9 ± 1.4^{b}	3.62 ± 0.04^{b}	3.7 ± 0.27^{b}	3.50 ± 0.06^{c}	5.4 ± 0.22^{b}	0.47 ± 0.06^{a}	0.9899 ± 0.0003^{c}	14.06 ± 0.02^{c}	20.8 ± 0.7^{a}	1.03 ± 0.23^{a}
**P**	18.8 ± 0.5^{c}	3.60 ± 0.08^{a}	4.14 ± 0.34^{b}	3.59 ± 0.07^{c}	4.95 ± 0.19^{b}	0.4 ± 0.00^{b}	0.9908 ± 0.0010^{c}	12.12 ± 0.80^{b}	17.6 ± 0.26^{b}	1.07 ± 1.25^{a}
**P2**	22.4 ± 0.9^{b}	3.47 ± 0.06^{a}	3.85 ± 0.15^{b}	3.38 ± 0.04^{b}	5.78 ± 0.22^{a}	0.53 ± 0.06^{a}	0.9896 ± 0.0009^{c}	13.85 ± 0.93^{c}	19.63 ± 0.42^{a}	0.27 ± 0.31^{a}
**VP**	22.1 ± 1.5^{b}	3.5 ± 0.05^{a}	4.92 ± 0.08^{a}	3.41 ± 0.01^{b}	5.4 ± 0.21^{b}	0.4 ± 0.00^{b}	0.9912 ± 0.0017^{b}	12.59 ± 1.79^{b}	19.87 ± 1.16^{a}	0.47 ± 0.64^{a}
**N**	21.9 ± 1.2^{b}	3.6 ± 0.06^{b}	3.73 ± 0.07^{b}	3.49 ± 0.04^{c}	5.33 ± 0.32^{b}	0.43 ± 0.06^{a}	0.9903 ± 0.0004^{c}	13.37 ± 0.51^{c}	19.9 ± 1.18^{a}	0.07 ± 0.12^{b}
**S**	23.0. ± 0.9^{c}	3.59 ± 0.02^{b}	4.07 ± 0.12^{b}	3.51 ± 0.05^{c}	5.47 ± 0.47^{a}	0.5 ± 0.00^{a}	0.9895 ± 0.0010^{c}	14.3 ± 0.72^{c}	20.5 ± 0.70^{a}	0.00 ± 0.00^{b}
**V**	23.4 ± 1.0^{b}	3.53 ± 0.07^{a}	4.97 ± 0.24^{a}	3.43 ± 0.10^{b}	5.82 ± 0.53^{a}	0.43 ± 0.06^{a}	0.9920 ± 0.0011^{b}	13.22 ± 1.20^{b}	23.83 ± 0.9^{b}	0.23 ± 0.25^{b}
**Z**	20.5 ± 1.5^{b}	3.33 ± 0.03^{b}	5.05 ± 0.27^{a}	3.27 ± 0.02^{a}	6.27 ± 0.27^{a}	0.43 ± 0.06^{a}	0.9921 ± 0.0013^{b}	12.26 ± 1.21^{b}	21.37 ± 0.67^{a}	0.03 ± 0.06^{b}

TA—total acidity; VA—volatile acidity; RD—relative density; TDE—total dry extract; RS—reducing sugars.

Table S3. Colour parameters of must and wine. Different letters in the same column indicate significant differences at $p < 0.05$. Lowercase letters are used for must and uppercase letters for wine.

Samples		L^*	a^*	b^*	Chroma	Hue
**Must**	D	38.9 ± 0.1[a]	0.1 ± 0.1[a]	3 ± 0.3[a]	3 ± 0.3[a]	88.7 ± 1.9[a]
	IJK	39.1 ± 0.5[a]	-0.3 ± 0.1[b]	3.1 ± 0.2[a]	3.1 ± 0.2[a]	96 ± 1.7[b]
	K	38.8 ± 0.6[a]	0.1 ± 0.1[a]	3.2 ± 0.2[a]	3.2 ± 0.2[a]	89 ± 2.8[b]
	P	40.9 ± 0.4[b]	0 ± 0.1[b]	3 ± 0.3[a]	3 ± 0.3[a]	89.1 ± 3[b]
	P2	39.7 ± 0.6[b]	0.1 ± 0.1[b]	3.1 ± 0.2[a]	3.1 ± 0.2[a]	88.7 ± 1.9[b]
	VP	43.6 ± 1.2[b]	-0.4 ± 0.2[a]	2.9 ± 0.2[a]	3 ± 0.2[a]	98 ± 3.2[a]
	**CSD**	**40.2 ± 1.8[b]**	**-0.1 ± 0.2[b]**	**3.1 ± 0.2[a]**	**3.1 ± 0.2[b]**	**91.6 ± 4.5[a]**
	N	39.2 ± 0.5[a]	-0.2 ± 0.2[b]	2.6 ± 0.1[b]	2.7 ± 0.1[b]	94 ± 4.4[b]
	S	41.1 ± 0.5[b]	0.1 ± 0[b]	2.8 ± 0.1[a]	2.8 ± 0.1[a]	87.7 ± 0.8[b]
	V	40.7 ± 0.2[b]	0.4 ± 0.1[a]	2.6 ± 0.3[a]	2.7 ± 0.3[a]	82.2 ± 1.5[a]
	Z	42.6 ± 1.1[b]	-0.1 ± 0.2[b]	3 ± 0.3[a]	3 ± 0.3[a]	92.6 ± 4.2[b]
	**ND**	**40.9 ± 1.4[b]**	**0.0 ± 0.3[b]**	**2.8 ± 0.2[a]**	**2.8 ± 0.2[a]**	**89.1 ± 5.6[b]**
**Wine**	D	42.7 ± 0.6[A]	-0.6 ± 0.1[A]	2 ± 0.2[A]	2 ± 0.2[A]	106.9 ± 4.5[A]
	IJK	43.3 ± 0.5[A]	-0.7 ± 0[A]	1.8 ± 0.2[A]	1.9 ± 0.2[A]	110.3 ± 2.8[A]
	K	43.7 ± 0.5[A]	-0.7 ± 0[A]	1.9 ± 0.1[A]	2 ± 0.1[A]	110.6 ± 1.9[A]
	P	43.7 ± 0.7[A]	-0.7 ± 0[A]	1.9 ± 0.2[A]	2 ± 0.2[A]	110 ± 1.3[A]
	P2	43 ± 0.6[A]	-0.6 ± 0[A]	2.1 ± 0.1[A]	2.2 ± 0.1[A]	106.1 ± 0.8[A]
	VP	45 ± 0.6[B]	-0.8 ± 0.2[A]	2.2 ± 0.4[A]	2.3 ± 0.4[A]	110.2 ± 7.6[A]
	**CSD**	**43.6 ± 0.9[A]**	**-0.7 ± 0.1[A]**	**2.0 ± 0.2[A]**	**2.1 ± 0.2[A]**	**109.0 ± 3.8[A]**
	N	42.7 ± 0.4[A]	-0.6 ± 0.1[A]	1.8 ± 0.1[A]	1.9 ± 0.1[A]	108.4 ± 2.7[A]
	S	45.2 ± 0.8[B]	-0.8 ± 0.1[A]	2.1 ± 0[A]	2.3 ± 0[A]	111.6 ± 2.2[A]
	V	46.9 ± 1.5[B]	-1.1 ± 0.2[B]	2.1 ± 0.1[A]	2.3 ± 0.1[A]	117.4 ± 5.4[B]
	Z	46.7 ± 0.1[B]	-1.2 ± 0.1[B]	2.0 ± 0.1[A]	2.3 ± 0.1[A]	120 ± 2.9[B]
	**ND**	**45.4 ± 1.9[B]**	**-0.9 ± 0.3[B]**	**2.0 ± 0.2[A]**	**2.2 ± 0.2[A]**	**114.3 ± 5.7[B]**

L*—lightness; a*—the range from green to red; b*—the range from blue to yellow; chroma—colour intensity; hue—colour changes from must to wine; ND—Northern Dalmatia; CSD—Central and Southern Dalmatia.
